# Anomalous Node Detection in Blockchain Networks Based on Graph Neural Networks

**DOI:** 10.3390/s25010001

**Published:** 2024-12-24

**Authors:** Ze Chang, Yunfei Cai, Xiao Fan Liu, Zhenping Xie, Yuan Liu, Qianyi Zhan

**Affiliations:** 1 School of Artificial Intelligence and Computer Science, Jiangnan University, Wuxi 214122, China; 6223115009@stu.jiangnan.edu.cn (Z.C.); 6233115008@stu.jiangnan.edu.cn (Y.C.); xiezp@jiangnan.edu.cn (Z.X.); lyuan1800@jiangnan.edu.cn (Y.L.); 2 Department of Media and Communication, City University of Hong Kong, Hong Kong SAR, China; xf.liu@cityu.edu.hk

**Keywords:** blockchain, graph neural network, ensemble learning, anomaly detection

## Abstract

With the rapid development of blockchain technology, fraudulent activities have significantly increased, posing a major threat to the personal assets of blockchain users. The blockchain transaction network formed during user transactions can be represented as a graph consisting of nodes and edges, making it suitable for a graph data structure. Fraudulent nodes in the transaction network are referred to as anomalous nodes. In recent years, the mainstream method for detecting anomalous nodes in graphs has been the use of graph data mining techniques. However, anomalous nodes typically constitute only a small portion of the transaction network, known as the minority class, while the majority of nodes are normal nodes, referred to as the majority class. This discrepancy in sample sizes results in class imbalance data, where models tend to overfit the features of the majority class and neglect those of the minority class. This issue presents significant challenges for traditional graph data mining techniques. In this paper, we propose a novel graph neural network method to overcome class imbalance issues by improving the Graph Attention Network (GAT) and incorporating ensemble learning concepts. Our method combines GAT with a subtree attention mechanism and two ensemble learning methods: Bootstrap Aggregating (Bagging) and Categorical Boosting (CAT), called SGAT-BC. We conducted experiments on four real-world blockchain transaction datasets, and the results demonstrate that SGAT-BC outperforms existing baseline models.

## 1. Introduction

In recent years, blockchain transaction technology has matured and developed, while being characterized by its decentralization and immutability [1]. These features ensure the transparency and security of transactions, providing a more efficient and secure solution for handling complex transactions [2]. However, blockchain transaction fraud has surged significantly due to its decentralized nature. According to the 2024 Chainalysis Blockchain Scam Report, the amount of money obtained by illicit addresses was USD 4.6 billion in 2018, reaching USD 24.2 billion by 2023 [3]. Blockchain fraud has caused substantial economic losses and poses a significant threat to the entire blockchain economic ecosystem. Therefore, detecting fraud nodes in blockchain transaction networks has become an important research topic and has garnered widespread attention.

In blockchain networks, transactions are recorded in the blockchain ledger, and each transaction can be traced back to its source and destination addresses [4]. Typically, fraudulent activities do not appear directly in transactions with the victim but rather conceal their identity and intent through more complex network structures [5]. In this intricate transfer process, fraudulent activities often conceal themselves within multi-level neighbor relationships in the transaction network. The transaction chain between the victim and the fraudster may involve multiple intermediary accounts [6]. These accounts appear legitimate but are actually involved in money laundering or fund transfers. Identifying fraud nodes requires aggregating multi-level neighbor information rather than just direct transaction relationships. Graph neural networks have a higher degree of information aggregation for direct neighbors, which makes detecting fraud nodes in blockchain transaction networks challenging.

Additionally, in the vast blockchain transaction network, normal transactions constitute the majority, while transactions involving fraud account for only a small proportion [7]. This data imbalance makes fraud detection even more difficult, as common machine learning algorithms may tend to overlook the minority class of fraudulent transactions when faced with severely imbalanced data [8].

Current methods for detecting fraudulent nodes in blockchain transaction networks primarily involve using graph neural networks (GNNs) to learn node representations and classify nodes [9]. Although GNNs possess strong capabilities in learning neighbor information from graph data [10], there is still room for improvement. Specifically, in aggregating multi-hop neighbor information and addressing data imbalance issues in the task of detecting fraudulent nodes in blockchain transaction networks.

In view of this, this paper proposes the combination of Enhanced GAT with ensemble learning. We combine the Graph Attention Network (GAT) with subtree attention to aggregate multi-hop neighbor information while retaining direct neighbor information. Then, we use the Bagging method to train multiple base classifiers and stack their predictions, with CAT serving as a meta-model for training. Finally, the meta-model provides the final prediction results. Our SGAT-BC model improves the accuracy and robustness of node detection, especially in tasks involving imbalanced datasets and learning complex structures in graph data.

The main contributions of this paper can be summarized as follows:

- We have designed a model that combines a Graph Attention Network (GAT) with subtree attention. While retaining direct neighbor information, it also learns multi-hop neighbor information to enhance the model’s ability to understand complex relationships. The introduction of subtree attention enables the model to identify potential anomalous nodes.
- We utilized the Integrated Bagging ensemble learning framework, dividing the data into multiple sub-training subsets. We then trained base classifiers on each subset separately and combined their predictions to obtain the final prediction.
- Traditional Bagging integrates the predictions of base classifiers through a voting mechanism. However, this method is overly simplistic and fails to effectively utilize the predictions of the base classifiers. Therefore, we applied the stacking approach to process the base classifiers’ predictions. Specifically, we used CAT as the meta-model and trained it using the predictions from the training and validation sets. The trained meta-model then provides the final predictions.

## 2. Related Work

In this section, we focus on two main aspects of anomaly detection in blockchain transaction networks: graph-based anomaly detection and imbalanced learning.

### 2.1. Graph-Based Anomaly Detection

Graph data structures can flexibly and intuitively capture the relationships and structures within data, making them suitable for many complex real-world scenarios. Among these, typical applications of anomaly detection include financial anomaly detection [11,12,13] and fraudulent review detection [14,15,16,17].

#### 2.1.1. Financial Anomaly Detection

GEM [11] is the first heterogeneous graph neural network designed for malicious account detection on the Alipay platform. Based on the heterogeneous relationship network between accounts and devices, GEM effectively distinguishes malicious account embeddings. However, GEM may face efficiency issues when handling large-scale data. SemiGNN [12] makes full use of both labeled and unlabeled data in multi-perspective data. It addresses the limitations of traditional methods in data utilization by considering the social relationships and diversity of users in financial services. Nevertheless, its robustness to noisy data may be insufficient. TTAGCN [13] proposes a temporal transaction aggregation network for phishing detection in the Ethereum network. By combining transaction features with statistical and structural features produced by graph neural networks, it enhances the recognition of phishing addresses. Yet, it may have limitations when dealing with dynamically changing network structures.

#### 2.1.2. Fraudulent Review Detection

FdGars [14] reveals the complexity of disguise and fraud by malicious accounts in online app stores, proposing an effective graph convolutional network method to identify these hidden malicious accounts. By integrating textual and behavioral features, it provides a new perspective on anti-spam and fraud detection. However, this method may be sensitive to the quality of data labeling. GraphConsis [15] addresses three major inconsistencies faced by graph neural networks in fraud detection: contextual, feature, and relational inconsistencies. It can maintain data diversity while filtering out irrelevant neighbors and accurately identifying fraudulent behaviors. But its performance may be limited on highly sparse graph data. CARE-GNN [16] uses reinforcement learning to find the optimal number of neighboring nodes, aggregating selected neighbors in different relationships to strengthen node representation. However, its training process is complex and computationally expensive.

Despite these significant achievements, the above methods still have limitations in handling large-scale and noisy data, making them less suitable for the demands of anomaly detection in blockchain transaction networks. Therefore, it is necessary to propose more effective methods to overcome these challenges.

### 2.2. Class Imbalance Learning

In real-world scenarios, most data are imbalanced, and thus, class imbalance classification has become a classical research direction in data mining. Research on class imbalance problems falls into three main directions: class imbalance classification based on resampling [18,19,20,21], class imbalance classification based on cost sensitivity [22,23,24], and class imbalance classification based on ensemble learning [25,26,27].

#### 2.2.1. Class Imbalance Classification Based on Resampling

Resampling methods can be divided into two types. One type is undersampling the majority class. Gupta et al. [18] used an undersampling algorithm to reduce the class imbalance issue in the data, improving the model’s accuracy in predicting the minority class. Peng et al. [19] addressed the shortcomings of undersampling strategies by parameterizing the sampler using meta-learning. However, undersampling still risks losing valuable information. The other type is oversampling the minority class. SMOTE [20] increases minority samples by generating new synthetic ones. GL-GAN [21] integrates the SMOTE method to explore local distributions in learned latent space and uses GANs to capture global information, generating minority class samples even in highly imbalanced scenarios.

However, undersampling may lead to the loss of valuable information from the majority class, affecting overall model performance. Oversampling methods like SMOTE increase minority samples by generating new synthetic ones but may introduce noise or lead to overfitting.

#### 2.2.2. Class Imbalance Classification Based on Cost Sensitivity

Cost-sensitive classification of class-imbalanced data takes the total misclassification costs for each class as the optimization objective, assigning higher misclassification costs to minority classes to achieve stronger performance. GAT-COBO [22] combines graph neural networks with cost-sensitive boosting. The embeddings learned in GAT are fed into a cost-sensitive learner, which adjusts weights according to misclassification costs, thereby enhancing the model’s focus on minority classes. Cui et al. [23] proposed a loss function based on effective sample numbers and used an innovative reweighting strategy to balance it. However, this method requires accurate setting of misclassification costs, which can be challenging in practical applications.

#### 2.2.3. Class Imbalance Classification Based on Ensemble Learning

Ensemble learning trains multiple base learners and combines their predictions according to a specific strategy to form a strong model. Vong et al. [25] introduced a new sequential ensemble learning framework that divides the majority samples into several small, disjoint subsets for training, making the framework less sensitive to highly imbalanced ratios. Guo et al. [26] proposed the ECPUT model, which builds constraint pairs from minority and majority samples and learns a projection matrix through these constraints, combining multiple base classifiers to improve minority class recognition. Ren et al. [27] proposed EASE, which generates balanced datasets for each base classifier, mitigating the negative effects of class imbalance on classifier performance. Liu et al. [28] introduced the EUS method, which samples multiple subsets from the majority class, trains multiple learners, and combines their outputs to iteratively train weak learners, removing correctly classified majority class samples in each round.

Compared to resampling and cost-sensitive methods, ensemble learning can effectively combine the advantages of multiple models, reducing the impact of class imbalance on model performance [29,30].

Therefore, we choose to adopt ensemble learning-based methods, aiming to improve the recognition of minority classes by training multiple models and integrating their predictions, which is more suitable for the requirements of anomaly detection in blockchain transaction networks.

## 3. Definition and Problem Statement

### 3.1. Definition

**Definition** **1** (Graph)**.**
*The transaction topology of blockchain can be defined as a normal graph model G=(V,X,A,E,Y), where $V=\{v_{1},v_{2},v_{3},\ldots ,v_{N}\}$ is a set of nodes. $X=\{x_{1},x_{2},x_{3},\ldots ,x_{N}\}$ is the set of node features, where $\;x_{i}\in R^{d}$ is the feature vector of node $\;v_{i}$. These vectors stacked into a matrix form the feature matrix $\;X\in R^{N\times d}$ of the graph G. $\;A\in R^{N\times N}$ represents the adjacency matrix of G, where $\;a_{i,j}$ = 1 indicates an edge between node $\;v_{i}$ and node $\;v_{j}$; otherwise, $\;a_{i,j}$ = 0, $\;E=\{e_{1},e_{2},e_{3},\ldots ,e_{M}\}$. $\;e_{j}=\left(v_{sj},v_{rj}\right)\in E$ is an edge between node $\;v_{s,j}$ and $\;v_{r,j}$, where $\;v_{s,j},v_{r,j}\in V$. $\;Y=\{y_{1},y_{2},y_{3},\ldots ,y_{N}\}$ is the set of labels corresponding to all nodes in the set V. For the convenience of representation, we encode the label $\;y_{i}$ as one-hot vector $\;y_{i}$.*

**Definition** **2** (Imbalanced Ratio)**.**
*Consider a collection of categories labeled as C, where $C_{1}$ and $C_{2}$ represent two distinct groups within C. The class imbalance ratio is denoted by IR and is calculated as the quotient of the size of $C_{1}$ to the size of $C_{2}$, represented as $IR=\frac{|C_{1}|}{|C_{2}|}$. The value of IR ranges from zero to infinity. When IR>1, it indicates that $C_{1}$ is the predominant category while $C_{2}$ is less common. Conversely, IR = 1 signals an equilibrium between the classes [31].*

### 3.2. Problem Statement

**Definition** **3** (Anomaly Detection in Graphs)**.**
*The task of identifying an anomaly within a graph is specified on an imbalanced graph structure, denoted as G=(V,X,A,E,Y), where the concept was initially introduced in Definition 2. Each node in V is categorized as either anomalous or legitimate. In our blockchain transaction data-formed graph, a node with a label of 1 indicates an anomaly, signifying involvement in illicit activities such as money laundering or fraud, while a label of 0 indicates a legitimate node. The main goal of anomaly detection using this graph structure is to isolate those nodes that are anomalous, standing out from the legitimate vertices, thus posing it as a problem of imbalanced node classification within a supervised learning framework on graph G.*

## 4. Methodology

### 4.1. Overview

In this section, we outline our novel methodological approach, which includes three primary steps: sampling the training set, training the base models, and obtaining the final output through the meta-model training.

Specifically, within the Bagging framework, we first sample k subsets from the training set and then train these subsets using our SGAT model (Section 4.2). Next, we consolidate the prediction results of these k base models on their respective training sets into a k-dimensional new feature, which is subsequently fed into the CAT framework (Section 4.3) to train a meta-model. Finally, the prediction results of these k base models on the test set are fed into the well-trained meta-model to produce the final prediction results.

### 4.2. SGAT Model

**Figure 1:**
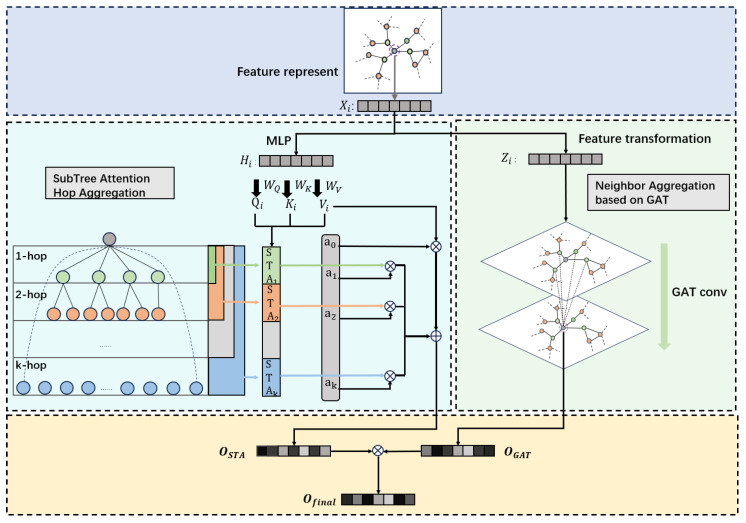
Overall framework of the SGAT model.

Our study introduces a novel model that combines Graph Attention Network (GAT) with subtree attention mechanism (STA) for anomaly detection in blockchain transaction data. As illustrated in Figure 1, this model effectively integrates the high sensitivity of GAT in processing direct neighbor information with the capability of STA to capture multi-hop neighbor information in deep graph structures. The attention mechanism of GAT [32] emphasizes the key features of direct neighbors, preventing excessive information dilution, while the STA module, inspired by previous advancements in adapting self-attention for graph structures [33], reveals indirect relationships and potential influence chains among multi-hop neighbor nodes, although important local features may be overlooked during information aggregation. Finally, a weighted fusion strategy using dot product achieves the optimal combination of the outputs of the two mechanisms, significantly improving the accuracy and sensitivity of anomaly detection in complex network environments.

#### 4.2.1. Multi-Hop Information Aggregation Based on Subtree Attention

In this subsection, we introduce the Multi-hop Information Aggregation based on subtree attention. This method uses subtree attention for message propagation. Initially, we use a multi-layer perceptron (MLP) [34] to process the initial feature vector x of a node, calculating the query, key, and value (QKV) values [35], which is described by the following equation:

(1) $$H=MLP\left(X\right),Q=H\left(W_{Q}\right),K=H\left(W_{K}\right),V=H\left(W_{V}\right)$$

The matrices $W_{Q}$, $W_{K}$, and $W_{V}$ are learnable projection matrices. The subtree attention mechanism uses queries, keys, and values as inputs to generate new values. The method for calculating the attention weights for the *k*th-hop neighbors is defined as $STA_{k}$. The weight calculation process for the *i*th node is represented as follows:

(2) $${\mathrm{STA}}_{k}{\left(Q,K,V\right)}_{i}:=\frac{\phi \left(Q_{i}\right)\cdot \sum_{j=1}^{N}{\hat{A}}_{ij}^{k}\left(\phi {\left(K_{j:}\right)}^{T}V_{j}\right)}{\phi \left(Q_{i}\right)\cdot \sum_{j=1}^{N}{\hat{A}}_{ij}^{k}\left(\phi {\left(K_{j:}\right)}^{T}\right)}$$

$STA_{k}{\left(QKV\right)}_{i}$ represents the attention computation of the subtree at the *k*-th level for node *i*, which corresponds to the *k*-hop neighbors. This computation utilizes a message passing mechanism, allowing keys and values to propagate along the edges. The algorithm operates with linear time complexity. The feature mapping employs a simple yet effective method introduced by [36], selecting ϕ(x)=elu(x)+1 as the feature mapping function. In Equation (2), message passing is implemented through two summations, $\sum_{j=1}^{N}{\hat{A}}_{ij}^{k}\left(\phi {\left(K_{j:}\right)}^{T}V_{j}\right)$ and $\sum_{j=1}^{N}{\hat{A}}_{ij}^{k}\left(\phi {\left(K_{j:}\right)}^{T}\right)$. Initially, $\phi (K_{i:})$ and $\phi {\left(K_{i:}\right)}^{T}V_{i:}$ are calculated for each node. These are then subjected to *k* steps of message passing. Ultimately, the aggregated keys and values $\sum_{j=1}^{N}{\hat{A}}_{ij}^{k}(\phi {\left(K_{j:}\right)}^{T}V_{j}$ and $\sum_{j=1}^{N}{\hat{A}}_{ij}^{k}(\phi {\left(K_{j:}\right)}^{T}$, along with the node’s own query $\phi (Q_{i})$, finalize the subtree attention computation. Finally, the representation of node *i* is determined by aggregating all layers of the subtree.

(3) $$\mathrm{STA}{\left(Q,K,V\right)}_{i}=\mathrm{AGGR}\left(\left\{\mathrm{STA}{\left(Q,K,V\right)}_{j}|j\in \left[1,K\right]\right\}\right)$$

This section employs an approach similar to Gaussian Process Regression for jump aggregation. Specifically, a learnable parameter $\beta_{k}$ (where k∈K, initially set to 1) is assigned to each jump. These parameters determine the weights with which the node aggregates information from each jump layer.

(4) $$O_{STA}=\sum_{k=0}^{K}\beta_{k}{\mathrm{STA}}_{k}\left(Q,K,V\right)$$

$O_{STA}$ represents the representation learned by the node based on the learnable weights and representations from various STA layers. In the left half of Figure 1, the aggregation process is observable. Through this process, the module can effectively learn more information from multi-hop neighbors. Moreover, thanks to the information propagation method of subtree attention, even if the majority of the nodes in the subtrees formed by two different nodes are the same, as long as their subtree structures differ, they can still gather different information through the subtree attention mechanism. However, although the message passing mechanism of subtree attention significantly reduces the issue of over-smoothing when aggregating multi-layer information, the information from direct neighbors might still be somewhat diminished in the context of anomaly detection.

#### 4.2.2. Neighbor Information Aggregation Based on GAT

In this module, we first process the initial feature vector *x* of the nodes through a feature transformation layer to enhance the expressive power of the nodes. The feature transformation process begins with the layer-by-layer processing of input features, with each layer’s output undergoing a nonlinear transformation through a ReLU activation function. The specific process is as follows: The initial input *x* is first processed by a MixedLinear layer, immediately followed by the application of a ReLU activation function to obtain the activated output. This output then serves as the input for the next Linear layer, and a ReLU activation function is applied again to this layer’s output. This process can be expressed by the following formula:

(5) $$z=\mathrm{ReLU}(W_{2}\cdot \mathrm{ReLU}\left(W_{1}\cdot x\right))$$

where $W_{1}$ and $W_{2}$ represent the weight matrices of the two layers. The output *z*, obtained after feature transformation, is used as the node embedding in GAT. Subsequently, the attention coefficients between adjacent nodes in the initial GAT can be expressed as follows:

(6) $$\alpha_{ij}=\frac{\exp(\mathrm{LeakyReLU}\left(a^{⊤}\left[Wz_{i}∥Wz_{j}\right]\right))}{\sum_{k\in N_{i}}\exp\left(\mathrm{LeakyReLU}\left(a^{⊤}\left[Wz_{i}∥Wz_{k}\right]\right)\right)}$$

Here, *a* represents the learnable attention function, implemented through a single-layer multi-layer perceptron (MLP). *W* is $R^{h\times d}$, a dimensional weight matrix used for linear transformation of node embeddings, where *h* is the number of neurons, a manually set model hyperparameter, and *d* is the dimension of *z*. The symbol ‖ denotes feature concatenation, and LeakyReLU() is the activation function. Thus, the new embedding $z_{i}^{\prime }$ of node *i* is calculated based on the attention coefficients and old embeddings $z_{i}$ as follows:

(7) $${\left(O_{\mathrm{GAT}}\right)}_{i}=z_{i}^{\prime }=\sigma \left(\sum_{j\in N_{i}}\alpha_{ij}Wz_{j}\right)$$

$N_{i}$ is the set of neighbors of node *i*, and σ is the activation function. To obtain richer representations, a multi-head attention mechanism can be employed as described in Equation (8)

(8) $${\left(O_{\mathrm{GAT}}\right)}_{i}=z_{i}^{\prime }={∥}_{q=1}^{Q}\sigma \left(\sum_{j\in N_{i}}\alpha_{ij}^{q}W^{q}z_{j}\right)$$

*Q* denotes the number of heads, and $\alpha_{ij}^{q}$ represents the attention coefficients of the *q*-th head.

We use $O_{\mathrm{GAT}}$ to represent the node representations after aggregation through the Graph Attention Network. Although the GAT excels in aggregating information from direct neighbors, it lacks proficiency in naturally aggregating information from multi-hop neighbors.

#### 4.2.3. Proposed SGAT

In Section 4.2.1, we introduce the subtree attention mechanism (STA). The STA module is designed to capture neighbor information in deep graph structures, which is crucial for understanding long-term patterns of node behavior. By learning information from multi-hop neighbors, STA can reveal indirect relationships and potential influence chains between nodes. However, focusing on deep structures might dilute direct neighbor information during aggregation because multi-hop aggregation may overshadow the features of close neighbors. This loss of information, especially in anomaly detection within complex networks, might lead to the oversight of critical local features, thereby affecting the sensitivity and accuracy of detection.

To address the limitations of STA in processing direct neighbor information, in Section 4.2.2, we introduce the Graph Attention Network (GAT). The GAT, through its attention mechanism, effectively weights the features of direct neighbors, thus highlighting the information most relevant to the target node. This allows the model to focus on direct, local node relationships without significant dilution. However, although the GAT excels at capturing direct neighbor information, it is not adept at handling multi-layer or multi-hop neighbor information, which may miss broader context understanding.

Therefore, by fusing the outputs of STA and GAT, our model can utilize the extensive information from multi-hop neighbors provided by STA while maintaining the high sensitivity of GAT to direct neighbors. This fusion method optimizes the capture and utilization of information without sacrificing any detail. Specifically, we adopted a fusion strategy by integrating the outputs of both networks through the dot product, defined as:

(9) $$O_{\mathrm{final}}=\mathrm{AGGR}\left(O_{\mathrm{STA}},O_{\mathrm{GAT}}\right)=\sum_{i=1}^{n}{\left(O_{\mathrm{STA}}\right)}_{i}\times {\left(O_{\mathrm{GAT}}\right)}_{i}$$

After aggregating the information from direct and multi-hop neighbors, we employ a composite loss function $L_{\mathrm{Total}}$, to optimize our model. The general form of the loss function is defined as follows:

(10) $$L=-\sum_{i=1}^{N}w_{i}\left[y_{i}\log\left({\hat{y}}_{i}\right)+\left(1-y_{i}\right)\log\left(1-{\hat{y}}_{i}\right)\right]$$

Subsequently, the composite loss function is defined as:

(11) $$L_{\mathrm{Total}}=\left(1-\gamma \right)\cdot L_{\mathrm{main}}+\gamma \cdot L_{\mathrm{GAT}}$$

where both $L_{\mathrm{main}}$ and $L_{\mathrm{GAT}}$ are computed using the same weighted cross-entropy loss function, corresponding to the outputs after aggregating with GAT and STA, respectively, and to the output of the GAT component. The choice of weighted cross-entropy loss is due to its ability to assign different weights to different categories, offering a solution to the issue of class imbalance present in our data.

Here, γ is a hyperparameter that balances the importance of the two model components during training, allowing us to adjust according to the specific requirements of the task. This combination strategy enables the model to learn representations for general features as well as complex relationships specific to the graph structure.

This not only enhances the model’s ability to detect potential relationships among multi-hop neighbors but also ensures the complete aggregation of information from direct neighbors, which is particularly important in anomaly detection tasks.

### 4.3. Ensemble Learning Algorithms

#### 4.3.1. Bootstrap Aggregating

**Figure 2:**
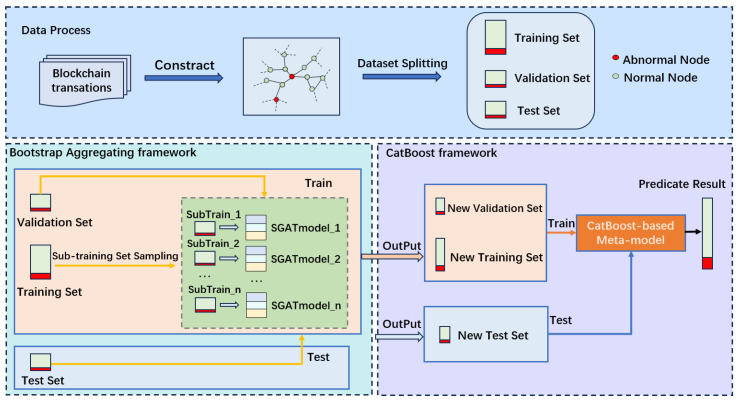
Overall framework of the hybrid ensemble learning model.

Ensemble learning is a machine learning method that improves the output quality by combining multiple models with imperfect performance, termed weak classifiers. When individual models operate under highly imbalanced node conditions, they are prone to biases, affecting both accuracy and reliability. To enhance the robustness of the model, ensemble learning aggregates the predictions of multiple base models, achieving higher precision and reliability. The overall framework of the Bagging method [37], as shown in the left half of Figure 2, involves dividing the training set into multiple subsets, maintaining the same node imbalance ratio as the original set. SubTrain sets are formed based on the ratio of normal to abnormal nodes in the training set. The total number of nodes *N* and the sampling percentage *p* define the total sampling size *n*:

(12) n=⌊N·p⌋

The number of samples for normal nodes $N_{\mathrm{normal}}$ and abnormal nodes $N_{\mathrm{abnormal}}$ are:

(13) $$\begin{array}{cc} n_{\mathrm{normal}} & =\lfloor N_{\mathrm{normal}}\cdot p\rfloor ,\\ n_{\mathrm{abnormal}} & =\lfloor N_{\mathrm{abnormal}}\cdot p\rfloor \end{array}$$

The sampling strategy includes separate sampling for normal and abnormal nodes, with resampling from the sampled nodes when necessary, to ensure each subset contains a sufficient number of abnormal nodes. The sampled node sets are denoted as $S_{\mathrm{normal}}$ and $S_{\mathrm{abnormal}}$, combined as:

(14) $$S_{\mathrm{SubTrainSet}}=S_{\mathrm{normal}}\cup S_{\mathrm{abnormal}}$$

Each SubTrain set independently trains an SGAT model, serving as a weak classifier in ensemble learning with consistent parameters, which then predicts the category of nodes in the test dataset and outputs a category probability vector. For each node *i* in the test set, we collect all the weak classifiers’ predictions to form a prediction matrix $P_{i}$, where $P_{i}\left[j,k\right]$ represents the probability that the *j*-th model predicts node *i* belongs to category *k*. For each node *i*, we construct a predicted category matrix *C* by selecting the category with the highest predicted probability.

(15) $$C_{i,j}=\arg\max_{k}P_{i}\left[j,k\right]$$

The final category prediction is obtained by majority voting over the predictions for all nodes. By integrating the predictions of multiple models, we can effectively reduce the random errors of individual models and enhance the robustness and accuracy for outlier categories in the dataset.

#### 4.3.2. Categorical Boosting

Through its efficient gradient boosting mechanism, CatBoost can quickly learn the complex relationships between outputs of different base models [38], thereby optimizing the overall prediction performance. In stacking ensemble, the predictions from various base models are typically used as new features input into the meta-model. CatBoost then utilizes these features for the final prediction in trainset and valset, which can significantly improve the model performance on complex and highly imbalanced datasets.

Specifically, after completing the independent training and prediction of each base model, to more effectively integrate this information, we do not adopt the traditional majority voting method. Instead, we implement an innovative stacking strategy using the CatBoost model to learn how to select the most probable category from these probability vectors. The CatBoost serves as the meta-model to further analyze and synthesize these prediction results.

For each node *i* in the training and validation sets, the prediction result *p* from each base model *j* is a probability vector, where $p_{i,j,k}$ represents the probability that model *j* predicts node *i* and belongs to category *k*. In this study, our nodes have two classifications: normal nodes, which constitute the vast majority, and abnormal nodes, which are a minority. Next, we use the predicted probabilities of the abnormal category (category 2) from each base model to construct the feature matrix. Let $p_{i,j,2}$ be the probability that model *j* predicts node *i* belongs to the second category. The feature matrices for the training set and validation set, $X_{\mathrm{train}}$ and $X_{\mathrm{val}}$, are, respectively:

(16) $$\begin{array}{cc} X_{\mathrm{train}} & =[p_{i,1,2},p_{i,2,2},\cdots ,p_{i,k,2}]\;\forall i\in \mathrm{train}\_\mathrm{mask}\\ X_{\mathrm{val}} & =[p_{i,1,2},p_{i,2,2},\cdots ,p_{i,k,2}]\;\forall i\in \mathrm{val}\_\mathrm{mask}\\ X_{\mathrm{test}} & =[p_{i,1,2},p_{i,2,2},\cdots ,p_{i,k,2}]\;\forall i\in \mathrm{test}\_\mathrm{mask} \end{array}$$

where *k* is the total number of base models. We use the feature vectors composed of the predictions from the training set and validation set as training data for the meta-model.

After the training of the meta-model is complete, we utilize the trained meta-model to make the final prediction on the feature matrix $X_{\mathrm{Test}}$ formed by the predictions of the base models on the test set $P_{\mathrm{test}}$. This method not only utilizes the diversity of the base models but also integrates the probability judgments of each category through the CatBoost.

## 5. Experiments

This study primarily addresses the following three questions:

- RQ1: What is the performance with respect to different training parameters?
- RQ2: Does the SGAT-BC model outperform the state-of-the-art methods for graph-based anomaly detection?
- RQ3: How do the key components contribute to the prediction?

### 5.1. Experimental Setup

#### 5.1.1. Dataset Description

In this section, we conduct anomaly detection experiments on four publicly available blockchain transaction datasets: Elliptic, AscendEXHacker, UpbitHack, and Ethereum transactions data. Below are the detailed descriptions of these datasets:

- The Elliptic Dataset is a well-known dataset extensively used for studying and analyzing the legality of Bitcoin transactions. Co-released by the IBM research team and Elliptic Company, the dataset categorizes nodes into legal, illegal, and unknown, with malicious activities such as extortion, money laundering, and scams classified as illegal transactions. It comprises 203,769 nodes and 234,355 edges, where nodes represent transactions and edges denote the flow of Bitcoin between these transactions. Among these nodes, 4545 are labeled as illegal (2%), 42,019 as legal (21%), and the remaining 157,205 are unlabeled. This results in a node imbalance ratio of 0.03. The dataset features 166 dimensions for each node, with the first 94 dimensions capturing transaction attributes such as time steps, in-degrees, out-degrees, transaction fees, and Bitcoin amounts. The remaining 72 dimensions represent aggregate features, summarizing the graph structure of a node’s direct neighbors. In this study, only the labeled nodes and the edges they form were selected for analysis, while all unlabeled nodes and their associated edges were removed. Due to intellectual property restrictions, the dataset provider has not disclosed detailed descriptions of all features, but a generalized overview of the publicly available features has been provided. To ensure consistency and scalability, numerical features were standardized based on their statistical properties, excluding specific non-numerical components.
- AscendEXHacker and UpbitHack are datasets published on XBlock, included in the EthereumHeist dataset. This dataset spans 2018 to 2022, focusing on representative theft cases on Ethereum and providing a robust foundation for blockchain anomaly detection research. We specifically selected two cases, AscendEXHacker and UpbitHack, as part of our study.
  For the AscendEXHacker dataset, the transaction graph comprises 6713 nodes (6646 normal nodes and 67 anomalous nodes) and 11,901 edges, with a node imbalance ratio (NodeIR) of 0.01, indicating a highly imbalanced class distribution. Similarly, the UpbitHack dataset contains a significantly larger graph with 568,994 nodes (559,250 normal nodes and 8744 anomalous nodes) and 1,447,348 edges, with a NodeIR of 0.03, reflecting a slight increase in anomaly representation but still posing challenges due to imbalance.
  The transaction graph is constructed from the raw transaction files using from and to fields to define sender and receiver relationships. Each node represents an Ethereum address, and edges denote transaction flows. Node labels are provided, with the label heist marking nodes involved in malicious activities, while normal nodes lack this label.
  Since the original data for both the AscendEXHacker and UpbitHack datasets lacks predefined features, we performed feature engineering to generate meaningful attributes for each node. First, we calculated the degree-related features, including the in-degree, out-degree, and total degree of each node, to capture the transactional relationships between nodes. Additionally, based on the raw transaction data, we extracted value-based features such as the mean transaction value (mean-value), maximum transaction value (max-value), and minimum transaction value (min-value). In Ethereum, gas represents the computational cost required to execute transactions or smart contracts. It ensures that users pay for the computational resources consumed, which prevents abuse of network resources. Gas-related features were also derived, including the mean gas price (mean-gasPrice), maximum gas price (max-gasPrice), and minimum gas price (min-gasPrice), as well as the mean gas used (mean-gasUsed), maximum gas used (max-gasUsed), and minimum gas used (min-gasUsed). These features reflect the computational cost and characteristics of the transactions associated with each node. Finally, we calculated the transaction frequency for each node by dividing its total degree by the time interval between the earliest and latest transactions. This comprehensive feature engineering process allowed us to enrich the dataset with node-level attributes, enabling more effective anomaly detection in Ethereum transaction networks.
- The Ethereum transactions dataset is an open-source blockchain transaction dataset available on GitHub. It consists of key attributes, including sender, receiver, amount, timestamp, fromIsPhi, and toIsPhi. A transaction graph is constructed from these data, where nodes represent entities (senders and receivers), and edges represent transactions between them. Labels are determined by the fromIsPhi and toIsPhi attributes, where fromIsPhi indicates that the sender is an anomaly node, and toIsPhi indicates that the receiver is an anomaly node.
  We first performed data cleaning to extract the nodes and edges for the graph. For feature engineering, we computed node-level attributes such as out-degree, in-degree, average degree, total degree, average sending amount, total sending amount, maximum sending amount, average receiving amount, total receiving amount, maximum receiving amount, transaction time interval ratio, and the total number of neighbors for each node. These engineered features were used to enrich the graph representation and provide comprehensive input for anomaly detection tasks. Finally, numerical features were standardized to ensure uniform scaling for downstream modeling tasks, and isolated or irrelevant nodes were removed from the transaction graph to retain only meaningful components.

Details of the node information, edge information, and the node imbalance ratio for these four datasets are provided in the Table 1.

#### 5.1.2. Compared Methods

To validate the effectiveness of our proposed method in detecting anomalies in imbalanced datasets based on graph neural networks, we compared it with several state-of-the-art graph neural network methods.

- **GCNs** [10]: Graph Convolutional Networks are a popular type of graph neural network that learns representations of nodes by propagating and transforming features across the nodes of a graph. GCNs utilize the adjacency matrix and node feature matrix of the graph to perform hierarchical feature extraction, capturing the local structural information of nodes.
- **GATs** [32]: Graph Attention Networks introduce an attention mechanism that dynamically determines the importance of different neighbors during the aggregation process of nodes. GATs can adaptively learn the weights between nodes, thus more effectively capturing the structural features of the graph.
- **Graphsage** [39]: Graph Sample and Aggregation (GraphSAGE) is an inductive learning framework that efficiently generates low-dimensional embeddings of nodes from large-scale graphs. GraphSAGE samples a fixed number of neighbors and uses aggregation functions, such as mean or pooling, to update the representation of nodes.
- **FdGars** [14]: FdGars employs a customized version of Graph Convolutional Networks (GCNs) to detect fraudulent accounts in online app store review systems. Unlike general-purpose GCNs, which primarily focus on hierarchical feature extraction from node features and adjacency matrices, the GCNs in FdGars are specifically optimized for anomaly detection tasks. The task-specific design enables FdGars to capture subtle relational and contextual features in the social graph, enhancing its capability to identify potential fraudulent activities.
- **Graphconsis** [15]: GraphConsis is a graph neural network framework designed for fraud detection. It addresses issues of inconsistency in graph models applied to fraud detection, such as contextual, feature, and relational inconsistencies. The framework integrates node features with contextual embeddings and designs a consistency score to filter out inconsistent neighbors based on their consistency scores, thereby defining ‘sampled nodes’ as those neighbors that meet a predefined threshold of consistency. This selection process ensures that only relevant and consistent information is used for learning relational attention weights.
- **CARE-GNN** [16]: CARE-GNN is an enhanced graph neural network approach for detecting disguised fraudsters in graph-structured data. It incorporates a novel attention mechanism and subgraph feature extraction strategy to identify and highlight areas of the graph that may be manipulated or influenced by disguised fraudsters. The method can learn complex fraud patterns and dynamically adjust its network structure to cope with the evolving strategies of fraudsters.
- **GAT-COBO** [22]: GAT-COBO is a cost-sensitive graph neural network model specifically designed for fraud detection in the telecommunications industry. This model integrates the capabilities of Graph Attention Networks (GATs) with cost-sensitive learning strategies to enhance performance in telecom fraud detection.
- **STAGNN** [33]: STAGNN is an enhanced graph neural network (GNN) model, specifically engineered for more efficient processing of graph-structured data. This model adaptively utilizes the root subtree structures within graphs to amplify its self-attention mechanisms, thereby enhancing both the performance and interpretability of the neural network across various tasks.
- **IForest** [40]: Isolation Forest is an ensemble-based algorithm specifically designed for anomaly detection. It isolates anomalies instead of profiling normal data points. By randomly selecting a feature and then randomly selecting a split value between the maximum and minimum values of the selected feature, Isolation Forest recursively partitions the data. Anomalies are expected to have shorter paths in the tree structure, thus isolating them efficiently.
- **LOF** [41]: Local Outlier Factor is an algorithm that measures the local deviation of a given data point with respect to its neighbors. It is based on a concept of local density, where locality is given by the k-nearest neighbors, whose distance is used to estimate the density. A point is considered as an outlier if the density around this point is significantly different from the density around its neighbors.
- **OCSVM** [42]: One-Class SVM is a specialized version of the SVM algorithm. It learns a decision function for anomaly detection by identifying the smallest region that encompasses the majority of the data points. Data points that do not fall within this region are considered anomalies.

#### 5.1.3. Evaluation Metrics

To comprehensively evaluate the performance of our proposed graph neural network-based anomaly detection method on imbalanced datasets, we employed various evaluation metrics that are pivotal for different aspects of predictive performance. These metrics are indispensable for providing a holistic assessment of the model’s ability to correctly identify the minority class anomalies, which is a critical requirement in anomaly detection tasks.

We employed four evaluation metrics as follows:

- **Macro Recall**: Measures the average proportion of actual anomalies that the model successfully identifies across all classes, treating each class equally regardless of its sample size. In anomaly detection, Macro Recall is particularly crucial as it ensures the model’s capability to detect rare and critical anomalies is not overshadowed by majority class performance.
- **Macro F1 Score**: The harmonic mean of macro precision and Macro Recall, offering a balanced evaluation of the model’s ability to identify anomalies correctly while maintaining robustness across all classes. This is especially important in anomaly detection, where a model must balance accuracy and coverage to effectively detect anomalies without being dominated by majority class performance.
- **Macro AUC**: The Macro Area Under the ROC Curve (AUC) provides an aggregate measure of the model’s ability to distinguish between classes across all thresholds, averaged over all classes. Macro AUC is advantageous in anomaly detection as it evaluates the model’s discriminative power in scenarios with significant class imbalance, ensuring fair assessment across both minority and majority classes.
- **G-Mean**: The geometric mean of the True Positive Rate (TPR) and the True Negative Rate (TNR), assessing the balance between the model’s sensitivity to the minority class and specificity to the majority class. In anomaly detection, G-Mean ensures that the model not only identifies anomalies effectively but also avoids excessive false positives, maintaining a balanced performance between critical minority and majority classes.

Overall, these four metrics provide complementary information from multiple aspects such as class distinction ability, minority class detection ability, and overall performance balance. This combination effectively avoids the bias that a single metric might introduce, thereby offering a comprehensive and reliable evaluation for anomaly detection tasks.

#### 5.1.4. Experiments Details

In the experiment, the training, validation, and test sets were allocated in the ratio of 60%, 20%, and 20%, respectively. While selecting training samples, we ensured that the proportion of normal and anomalous nodes in the training set remained consistent with the entire dataset. For our proposed SGAT-BC method, we set the embedding size of the hidden layer (64), learning rate (0.002), attention weight (0.6), number of epochs (3000), and height of subtree (3). For all comparison methods, we set the parameters according to their official implementations. All models were run in Python 3.9, using one GeForce RTX 3090 GPU and 32GB RAM.

### 5.2. Sensitivity Analysis (RQ1)

**Figure 3:**
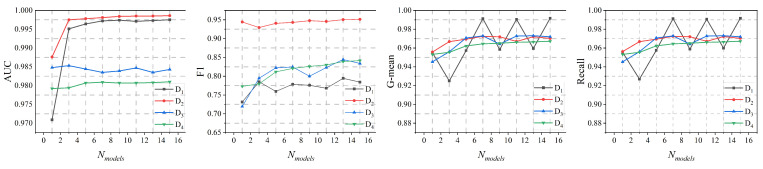
Sensitivity analysis of SGAT-BC with different numbers of base models.

To address RQ1, we further evaluated the performance of SGAT-BC in terms of both the number of base models and the size of the training set. Figure 3 illustrates the performance of SGAT-BC on four datasets under different numbers of base models. Specifically, we observe the following: (1) With an increase in the number of base models, the overall performance of the model gradually improves, as seen from the comprehensive evaluation across all datasets. For example, when increasing the number of base models from 1 to 16, the AUC on D1 improves from approximately 0.9709 to about 0.9975, while D2 consistently maintains near-perfect AUC values around 0.998. (2) Except for $D_{1}$, the performance on the remaining datasets remains relatively stable, with minor fluctuations. $D_{1}$ exhibits significant fluctuations due to its unique characteristics, namely, a small data size and a high absolute number of anomalous nodes, leading to substantial variability in response to parameter changes. However, even under such fluctuations, the performance shows an overall upward trend. Notably, even at D1’s lowest performance point, the AUC remains above 0.970.

**Figure 4:**
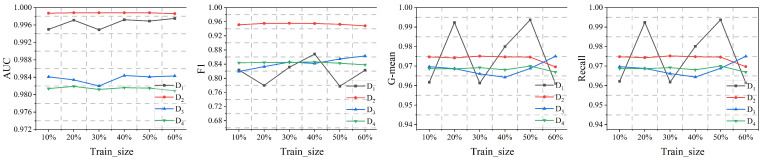
Sensitivity analysis of SGAT-BC with different train sizes.

Through Figure 4, we observe that, apart from $D_{1}$, the performance on the other three datasets remains relatively stable. The peculiarities of $D_{1}$ result in considerable fluctuations, yet even at its lowest performance point, there is a substantial improvement compared to the baseline models.

### 5.3. Performance Comparison (RQ2)

Table 2 and Table 3 present the performance of our proposed model, SGAT-BC, compared with various baseline models on four blockchain transaction datasets ($D_{1}$, $D_{2}$, $D_{3}$, and $D_{4}$) in terms of Macro AUC, Macro F1, Macro Recall, and G-Mean. For ease of discussion, we categorize the baseline models into three groups: general GNN models (e.g., GCN, GAT, GraphSage, STAGNN), GNN models specifically designed for fraud detection (e.g., GAT-COBO, CARE-GNN, GraphConsis, FdGars, Player2vec), and traditional anomaly detection methods (e.g., IForest, LOF, OCSVM).

As shown in Table 2 and Table 3, SGAT-BC maintains a leading advantage across all datasets and metrics. For example, on the $D_{1}$ dataset, SGAT-BC achieves a Macro AUC of 0.9979. Compared to the next best-performing general GNN model (e.g., GAT with 0.9567), this is about a 4.3% improvement. Relative to the best-performing general GNN baseline (e.g., STAGNN with 0.7038), the improvement exceeds 29%, and compared to a traditional anomaly detection method (e.g., IForest with 0.0167), the performance gain is two orders of magnitude. These substantial improvements also hold true for other metrics (Macro F1, Macro Recall, and G-Mean).

The class imbalance ratio in $D_{3}$ (approximately 0.11) is slightly less severe than in the other datasets, allowing general models to perform somewhat better on $D_{3}$ (for instance, GCN achieves a Macro Recall of 0.6073 on $D_{3}$). However, when confronted with more severely imbalanced datasets such as $D_{2}$ and $D_{4}$, most models experience a noticeable drop in Macro Recall and G-Mean. For example, GraphSage’s G-Mean is only 0.0655 on $D_{2}$ and even lower on $D_{4}$. This indicates that conventional models struggle to maintain stable anomaly detection capabilities under extreme imbalance. In contrast, SGAT-BC still achieves high Macro Recall and G-Mean on $D_{2}$ and $D_{4}$—both above 0.98 on $D_{4}$—outperforming other models by tens of percentage points. This strongly demonstrates the effectiveness and robustness of our ensemble learning strategy and imbalance handling mechanism in real-world scenarios.

STAGNN, as a relatively strong model among general GNNs due to its ability to leverage multi-hop neighborhood information, still fails to effectively address the challenges posed by imbalanced data. Consequently, it falls significantly behind SGAT-BC on more challenging datasets like $D_{2}$ and $D_{4}$. SGAT-BC not only exploits multi-hop information but also integrates an improved Bagging ensemble learning algorithm, thereby consistently improving metrics related to model balance (such as Macro Recall and G-Mean). This suggests that our approach does not rely solely on richer neighborhood information; rather, it simultaneously focuses on fusion strategies and imbalance handling to achieve a comprehensive performance boost.

Among the GNN models specifically designed for fraud detection, GAT-COBO stands out as comparatively strong, often ranking second behind SGAT-BC on $D_{1}$, $D_{2}$, and $D_{3}$. This can be attributed to its weighted ensemble method, which provides some adaptability to imbalanced data. However, SGAT-BC still surpasses GAT-COBO by more than 2–5% in multiple metrics. For instance, on $D_{1}$, SGAT-BC achieves a Macro F1 of 0.9276, compared to GAT-COBO’s 0.5372—an improvement of nearly 73%. On $D_{2}$, SGAT-BC attains a G-Mean of approximately 0.9853 versus GAT-COBO’s 0.7167, exceeding a 30 percentage-point improvement. This shows that our strategy is more comprehensive and effective in handling complex imbalance and graph structures.

Traditional anomaly detection methods (IForest, LOF, OCSVM) perform very poorly on graph-structured data. For example, on $D_{1}$, IForest achieves a Macro AUC of only 0.0167 and a Macro Recall of 0.9394. Although the Macro Recall appears high, the Macro F1 score is extremely low, indicating that under severe imbalance these methods cannot simultaneously maintain precision and recall. In contrast, SGAT-BC achieves a balanced performance with high Macro AUC, Macro F1, and Macro Recall on the same dataset. Such balance is crucial in real-world anomaly detection: one cannot simply capture the few positive anomalies at the expense of overall precision, nor can precision be maintained only by sacrificing recall.

In conclusion, the results and analyses convey a clear message: In complex graph-structured and highly imbalanced datasets, SGAT-BC—by leveraging subtree-level attention for deep feature extraction combined with GAT, alongside Bagging and CatBoost ensemble strategies—establishes a robust anomaly detection framework that not only enhances all evaluation metrics but also demonstrates strong stability. Whether compared to general GNNs, specialized fraud detection GNNs, or traditional anomaly detection methods, SGAT-BC’s substantial and comprehensive improvements in Macro AUC, Macro F1, Macro Recall, and G-Mean serve as compelling evidence for its practicality and broad applicability.

### 5.4. Ablation Study (RQ3)

To answer RQ3 and validate the effectiveness of our innovation, we identified three key components of SGAT-BC and individually eliminated these components, which are the subtree attention mechanism module (SGAT-BC/s), Bagging ensemble learning module (SGAT-BC/b), and CAT stacking module (SGAT-BC/c). Through Table 4 and Table 5, we can observe the following aspects:

- Compared with SGAT-BC, the performance of SGAT-BC/s decreased on all datasets across four evaluation metrics, especially on Recall and G-Mean. On the $D_{1}$ and $D_{2}$, Recall decreased by 6.63% and 7%, respectively, while G-Mean decreased by 6.86% and 7.27%, respectively. This is because the aggregation of multi-hop neighbor information by STA allows the model to better identify anomalous nodes, achieving better performance in both identifying anomalous and normal nodes.
- After removing the Bagging ensemble learning framework, SGAT-BC/b only maintained its performance on the F1 metric of the ETD dataset. However, it decreased across all evaluation metrics on all other datasets. On the $D_{3}$, AUC, F1, Recall, and G-Mean showed the most significant declines, decreasing by 1.85%, 32.6%, 6.05%, and 15.38%, respectively. This is because in blockchain anomaly detection tasks, the datasets are highly imbalanced. Removing the Bagging ensemble learning framework results in training only a single model for evaluation, which causes the model to be biased toward the majority class, leading to decreased recognition ability for minority classes. Furthermore, due to dataset imbalance, training a single model increases model bias and reduces robustness.
- After removing the CAT stacking module to obtain SGAT-BC/c, we found that SGAT-BC/c showed the most significant decreases in F1, Recall, and G-Mean on the $D_{4}$, decreasing by 4.41%, 3.05%, and 3.08%, respectively. Traditional Bagging ensemble learning handles base model predictions too directly by using a simple voting method. This approach cannot accurately combine the predictions of all base models. Instead, we use CAT as a meta-model to train on the output predictions of all base models, which better leverages the training results of the base models.

Overall, the performance of the complete SGAT-BC on these four datasets is the best, which demonstrates the positive role of all three key components of the model. Under the influence of these three key components, the model exhibits good performance in both accuracy and robustness for anomaly detection in imbalanced datasets.

## 6. Conclusions

### 6.1. Discussion

The class imbalance problem in graph data significantly impacts blockchain anomaly node detection, yet it has often been overlooked in previous research. This paper presents a novel ensemble learning method based on graph neural networks (GNNs) to address this challenge. Specifically, we utilize an improved SGAT as the base classifier and employ the Bagging ensemble learning algorithm to train multiple base classifiers. Subsequently, the predictions from these base classifiers are input into the CAT meta-model for stacking integration, thereby producing the final classification results.

In experiments conducted on four real-world blockchain transaction datasets, SGAT-BC significantly outperforms general GNN models (such as GCN, GAT, GraphSage, STAGNN), GNN models specifically designed for fraud detection (such as GAT-COBO), and traditional anomaly detection methods (such as IForest, LOF, OCSVM) across metrics including Macro AUC, Macro F1, Macro Recall, and G-Mean. Notably, in datasets with severe class imbalance, SGAT-BC still achieves high Recall and G-Mean values above 0.98, a performance level that other methods cannot match. This improvement is not merely incremental but represents a fundamental breakthrough in maintaining stable and balanced detection performance under extremely imbalanced conditions.

The results demonstrate that SGAT-BC does not rely solely on the simple utilization of multi-hop neighborhood information as in STAGNN. Instead, by integrating an improved Bagging ensemble strategy with a robust meta-model design, SGAT-BC effectively mitigates the performance fluctuations commonly observed in other methods. In this approach, enhancing Recall does not come at the expense of precision, ensuring a comprehensive balance in detection performance. This balance is crucial for practical anomaly detection applications, where capturing rare anomalies while minimizing false alarms is essential. SGAT-BC’s performance provides strong support for achieving precise and stable anomaly detection in complex, imbalanced scenarios.

### 6.2. Future Work

In addition to its excellent experimental performance, the design of SGAT-BC also offers insights for future research directions. The experimental results indicate that not only should richer graph representations be considered, but also the active incorporation of ensemble learning principles to achieve more robust and scalable solutions to the imbalance problem. These findings emphasize the necessity of organically integrating graph representation learning with imbalance handling strategies, providing a solid foundation for expanding applications beyond blockchain; for example, SGAT-BC can be applied in telecom fraud detection, social network scams, and financial anomaly analysis.

Future work could explore more dynamic ensemble methods, incorporate advanced sampling or cost-sensitive learning strategies, and consider introducing richer feature information (such as time-series features and semantic attributes) to enhance the adaptability and interpretability of SGAT-BC. Additionally, transferring imbalance handling techniques from non-graph domains to the GNN framework and testing the generalizability of SGAT-BC in a wider range of real-world scenarios will help further validate and refine this method, thereby solidifying its position in increasingly complex and diverse imbalance problem environments. Furthermore, future research can delve deeper into performance optimizations, such as time measures and comparative analyses.

## Figures and Tables

**Table 1:** DataSet information.

DataSet	Normal Node	Anomalous Node	NodeIR	Edge
AscendEXHacker ($D_{1}$)	6646	67	0.01	11,901
Ethereum transactions data ($D_{2}$)	45,782	1165	0.03	103,035
Elliptic ($D_{3}$)	42,019	4545	0.11	101,188
UpbitHack ($D_{4}$)	559,250	8744	0.03	1,447,348

**Table 2:** Performance comparison results with regard to Macro AUC, Macro F1, Macro Recall, and G-Mean on $D_{1}$ and $D_{2}$ datasets.The Bolded results present the highest performance.

Method	$D_{1}$				$D_{2}$			
	Macro AUC	Macro F1	Macro Recall	G-Mean	Macro AUC	Macro F1	Macro Recall	G-Mean
GCN	0.9839	0.6313	0.6102	0.4733	0.9093	0.6220	0.5737	0.3844
GAT	0.9567	0.7815	0.8584	0.8479	0.9403	0.7432	0.6947	0.6263
Graphsage	0.9346	0.4976	0.5000	0.0000	0.8287	0.4980	0.5021	0.0655
FdGars	0.4739	0.5278	0.6434	0.5726	0.5993	0.0807	0.4979	0.2372
Player2vec	0.1874	0.1119	0.4083	0.2857	0.3498	0.0735	0.5016	0.2220
GraphConsis	0.8627	0.5340	0.5340	0.2761	0.9598	0.5563	0.5353	0.2698
CARE-GNN	0.8727	0.2209	0.5973	0.4938	0.9516	0.6693	0.7908	0.7745
GAT-COBO	0.5533	0.5372	0.7253	0.6994	0.9648	0.7600	0.7539	0.7167
STAGNN	0.7038	0.7470	0.7233	0.7470	0.9169	0.7663	0.8241	0.7663
IForest	0.0167	0.5640	0.9394	0.9389	0.0370	0.6503	0.8905	0.8900
LOF	0.6466	0.5024	0.6153	0.5443	0.7345	0.5188	0.5746	0.4710
OCSVM	0.0172	0.5648	0.9398	0.9393	0.2827	0.4994	0.7458	0.7454
SGATBC	**0.9979**	**0.9276**	**0.9981**	**0.9981**	**0.982**	**0.8722**	**0.9853**	**0.9853**

**Table 3:** Performance comparison results with regard to Macro AUC, Macro F1, Macro Recall, and G-Mean on $D_{3}$ and $D_{4}$ datasets. The Bolded results present the highest performance.

Method	$D_{3}$				$D_{3}$			
	Macro AUC	Macro F1	Macro Recall	G-Mean	Macro AUC	Macro F1	Macro Recall	G-Mean
GCN	0.8752	0.6506	0.6073	0.4686	0.9648	0.7730	0.6993	0.6319
GAT	0.9218	0.7279	0.6847	0.6171	0.9553	0.7420	0.6691	0.5821
Graphsage	0.9382	0.8632	0.8205	0.8026	…	…	…	…
FdGars	0.4136	0.4434	0.5734	0.5722	0.4201	0.1942	0.5398	0.4122
Player2vec	0.5331	0.2062	0.5239	0.3453	0.5189	0.1818	0.5310	0.3939
GraphConsis	0.6977	0.4752	0.4985	0.0468	…	…	…	…
CARE-GNN	0.9113	0.6190	0.8078	0.8036	0.9577	0.6276	0.9117	0.9107
GAT-COBO	0.9749	0.9081	0.8737	0.8652	0.9725	0.7978	0.9372	0.9368
STAGNN	0.9308	0.9301	0.9305	0.9301	0.4839	0.5000	0.4918	0.5000
IForest	0.9013	0.4453	0.4447	0.0140	0.0854	0.6356	0.7854	0.7741
LOF	0.4429	0.5113	0.5114	0.3298	0.5434	0.4770	0.4729	0.2069
OCSVM	0.8418	0.4537	0.4532	0.1180	0.3261	0.5934	0.7022	0.6697
SGATBC	**0.9994**	**0.9933**	**0.9962**	**0.9962**	**0.9811**	**0.9811**	**0.9811**	**0.9811**

**Table 4:** Ablation results for $D_{1}$ and $D_{2}$ datasets.

Method	$D_{1}$				$D_{2}$			
	Macro AUC	Macro F1	Macro Recall	G-Mean	Macro AUC	Macro F1	Macro Recall	G-Mean
SGATBC	0.9979	**0.9276**	**0.9981**	**0.9981**	0.9820	0.8722	**0.9853**	**0.9853**
SGAT-BC∖STA	0.9975	0.9051	0.9318	0.9295	0.9806	0.8490	0.9153	0.9126
SGAT-BC∖CAT	**0.9982**	0.8305	0.9944	0.9943	**0.9824**	0.8861	0.8972	0.8921
SGAT-BC∖Bagging	0.9938	0.8125	0.9777	0.9774	0.9797	**0.8928**	0.8938	0.8904

**Table 5:** Ablation results for $D_{3}$ and $D_{4}$ datasets.

Method	$D_{3}$				$D_{4}$			
	Macro AUC	Macro F1	Macro Recall	G-Mean	Macro AUC	Macro F1	Macro Recall	G-Mean
SGATBC	**0.9994**	**0.9933**	**0.9962**	**0.9962**	**0.9811**	**0.8707**	**0.9761**	**0.9761**
SGAT-BC∖STA	0.9981	0.9783	0.9940	0.9940	0.9782	0.8557	0.9654	0.9654
SGAT-BC∖CAT	0.9994	0.9639	0.9907	0.9907	0.9808	0.8266	0.9456	0.9453
SGAT-BC∖Bagging	0.9809	0.6673	0.9357	0.8424	0.9789	0.8213	0.9446	0.9421

## Data Availability

The datasets used in this study can be found at the following links: Elliptic dataset: https://www.kaggle.com/datasets/ellipticco/elliptic-data-set/ accessed on 18 December 2024, AscendEXHacker and UpbitHack datasets: https://www.dropbox.com/scl/fo/ayk5juz7wn5q82o1dlet3/AC8FHG2bjOafiGmGu9W22kc?rlkey=zc1rhb1xtzvtdqwe3mee1zick&e=3 accessed on 18 December 2024, Ethereum transactions dataset: https://github.com/Swarnajit21/phishing_scam_detector_1907/blob/main/data.csv accessed on 18 December 2024.
